# Musculoskeletal Findings in Behcet's Disease

**DOI:** 10.1155/2012/653806

**Published:** 2011-09-25

**Authors:** Ali Bicer

**Affiliations:** Department of Physical Medicine and Rehabilitation, Faculty of Medicine, Mersin University, Zeytinlibahce Street, 33079 Mersin, Turkey

## Abstract

Behcet's disease is a multisystem disease characterized by recurrent oral and genital ulcers, relapsing uveitis, mucocutaneous, articular, gastrointestinal, neurologic, and vascular manifestations. Rheumatologic manifestations may also occur in Behcet's disease, and arthritis and arthralgia are the most common musculoskeletal findings followed by enthesopathy, avascular necrosis, myalgia, and myositis. Although the main pathology of Behcet's disease has been known to be the underlying vasculitis, the etiology and exact pathogenesis of the disease are still unclear. Musculoskeletal findings of Behcet's disease, the relationship between Behcet's disease and spondyloarthropathy disease complex, and the status of bone metabolism in patients with Behcet's disease were discussed in this paper.

## 1. Introduction

Behcet's disease (BD) is a chronic and recurrent multisystemic disorder, mainly characterized by oral and genital aphthous ulcers, skin lesions, and uveitis, occasionally resulting from inflammation in central nervous and gastrointestinal system, lungs, kidneys, and joints [1]. Additionally, BD is described as a systemic, vasculitic disorder in which both arteries and veins of any kind of organ may involve [2]. The disease may be distributed worldwide, but it is more prevalent in Asian and Mediterranean regions [3].

The etiology and pathogenesis of BD still remain obscure. However, both genetic and environmental factors, such as microbial pathogens, have been proposed to initiate a congenital and/or acquired immune dysfunction that cause development of the clinical manifestations of the disease [4]. Musculoskeletal involvement in BD is one of the most frequent findings among the clinical manifestations.  Table 1 summarizes the musculoskeletal findings of BD, found in the medical literature. Arthritis and arthralgia are the most common musculoskeletal findings of the disease [5]. Although the frequency of the arthritis has been increasing, it has not been yet included in any International Study Group (ISG) Criteria of BD [6]. Furthermore, it was also claimed that the disease could be classified into the spondyloarthropathy (SpA) complex, because of the presence of sacroiliitis in BD and the clinical overlap between BD and some disease of the SpA complex [7]. On the other hand, the chronicity and factors involved in inflammatory process of the disease and the drugs used for the treatment of BD might have negative effects on bone remodeling, consequently resulting in osteoporosis in BD [8].

In this paper, musculoskeletal findings of BD, the relationship between BD and SpA disease complex, and osteoporosis were discussed.

## 2. Arthritis

Arthritis and arthralgia are the most common rheumatologic findings in BD and their prevalence varies between 40 and 70% [1, 5, 9]. Subjects with arthritis in BD predominantly present with recurrent, self-limited, nondeforming and nonerosive, inflammatory asymmetric mono-oligoarthritis, affecting most frequently the larger joints suck, as knees, wrists, ankles and elbows [1, 5, 6, 9, 10]. Erosive forms of arthritis in BD have been reported in only small number of cases, and the most effected locations are axial joint (sacroiliac), enthesis (calcaneal), and peripheral joints such as metatarsophalangeal and interphalangeal joints of the feet and intercarpal and metacarpophalangeal joints of the hand, wrist and knee [11, 12]. Clinical hand involvement in BD was investigated extensively in a study where the prevalence of hand involvement in the disease was found to be high [13]. They also found that the terminal phalangeal tuft resorption that might be related to a specific pattern, induced by the vasculitic process due to the repeated digital infarcts, and the rheumatoid-like hand findings were the most frequent hands in patients with BD. 

The mechanism of arthritis is not fully understood, but inflammation and hyperemia (synovitis) are tried to be explained by vasculitis that constitutes the primary pathology in BD [23]. Synovial inflammation has been demonstrated in various reports where hypertrophy and hyperplasia of synovial lining cells, hypervascularity, subsynovial accumulation of inflammatory cells, and replacement of superficial zones of the synovial membrane by dense inflamed granulation tissue were described, without involvement of the deeper layers [5, 9, 24]. However, Moll et al. suggested that synovitis was characterized by large areas of hyperemic synovitis without villi or a distinctive vascular pattern in early untreated BD [25]. Cañete et al. stated that the polymorphonuclear neutrophil and lymphocytes, as important infiltrating cell populations, were found to be the indicators of cytotoxic molecules in early untreated synovitis in BD [26]. The authors also claimed that no evidence for neutrophilic vasculitis and no any other form of vessel pathology in the synovial pathology were detected.

Although there is no clear consensus on its etiology in terms of autoimmunity, some authors have declared that BD could be considered as an autoinflammatory disease due to the fact that both diseases may have the similar clinical characteristics [27]. However, investigations showed no evidence of increase in antibody concentration and T-cell-specific antigens in BD [28, 29]. 

BD is mostly diagnosed according to ISG Criteria for BD [30]. Although arthritis is seen in more than half of the subjects, it has not been considered among the ISG Criteria for BD. A possible explanation for this exception is that complaints about joints in society are very common, and, therefore, investigators ignore to consider these complaints or findings among the symptoms of the disease [6]. Since the laboratory findings in BD are nonspecific, its diagnosis is based exclusively on clinical evaluation of subjects. A detailed clinical history and classic manifestation of nonerosive arthritis on physical examination is diagnostic for BD. However, the presence of destructive lesions may confuse the diagnosis of BD and lead to investigate for another cause of destructive lesions, such as rheumatoid arthritis, psoriatic arthritis, and ankylosing spondylitis (AS) [23]. In vast majority of the patients with BD, colchicine (1-2 mg/day) is the drug of choice for the management of nonerosive arthritis [5]. However, destructive arthritis covers a broad spectrum of therapeutic alternatives, including colchicine and nonsteroidal anti-inflammatory drugs at first-line treatment, local corticosteroid injections, and low-dose systemic corticosteroids [10, 23]. Azathioprine and tumor necrosis factor-alpha (TNF-*α*) blockers may have some beneficial effects in rare cases with resistant, longer-lasting, and disabling conditions [31].

## 3. Spondyloarthropathy and Behcet's Disease

One of the remarkable questions in the past was the inclusion of BD in SpA disease complex. Since some investigators have reported the high prevalence of sacroiliitis associated to BD, the coexistence of BD and AS, and the presence of clinical overlap between BD and some subgroup of SpA disease complex such as inflammatory bowel disease and Reiter's syndrome, they claimed that BD could be included in seronegative SpA group [7, 32, 33]. The issue of sacroiliitis associated with BD had been exclusively investigated, and the prevalence of sacroiliitis in BD was highly variable. Yazici et al. have stated that high prevalence of sacroiliitis, found in studies, may be due to the fact that most of the studies had no control groups. Moreover, they claimed that intra- and interobserver variability in interpreting the AP pelvis radiographs is high [34]. Some authors have found that the prevalence of sacroiliitis has not been different from that in general population [15, 34].

The diseases in SpA group have some similar common features. One of the most striking associations among the clinical characteristics in SpA complex is with the enthesitis, HLA-B27 antigen, eye involvement [7, 35]. Although there have been conflicting results for the prevalence of HLA-B27 in patients with BD, BD seems to be associated with HLA-B51 rather than HLA-B27 [7, 36]. There are also some reports regarding the presence of acne-associated arthritis in a subgroup of patients with BD that may be considered within the broader concept of SpA disease complex [37]. Furthermore, Hatemi et al. have stated that the increased presence of enthesopathy in BD who also have acne and arthritis compared with that BD without arthritis supported the hypothesis that the BD having also acne and arthritis form a distinct cluster [35]. Findings of investigations regarding the prevalence of enthesopathy in BD are inconsistent. The prevalence of enthesopathy has been found to be higher (38%) in some clinical trials [9, 18], but lower rates (3.4%) has also been reported [7]. This wide variation in the reported frequency in BD was explained by the fact that radiography and physical examination were not sensitive methods for detecting enthesopathy and the differences in selected study groups [35]. On the other hand, the pattern of eye involvement is known to be different between SpA and BD. The uveitis of SpA mostly is in benign character, whereas uveitis in BD occasionally results in loss of vision [38]. 

Conclusively, the lack of clear association of HLA-B27 and BD, the disparity of the eye involvement in SpA and BD, and conflict reports on sacroiliitis and enthesitis in BD have complicated the enrollment of the BD into SpA disease complex.

## 4. Bone Metabolism

The chronicity and vasculitic background of the disease and the drugs for the treatment of BD suggest that bone metabolism might be affected negatively, which then leads to bone loss, resulting in osteoporosis in patients with BD [8]. Osteoporosis is a common disease characterized by a systemic impairment of bone mass and microarchitecture that causes fragility fractures [39]. The pathogenesis of osteoporosis is mainly considered as an imbalance of the remodeling process, and inflammatory molecules, such as proinflammatory cytokines, interleukins, and TNF-*α* play an imported role to regulate bone metabolism [39, 40]. On the other hand, the primer pathology in BD is inflammatory process of small arteries and veins and thrombosis as a result of vasculitis of vasa vasorum [41]. Elevated levels of immunoglobulins, immune complexes, and complement and acute-phase proteins reveal that the immune system is also involved and activated in the disease process [42]. Investigations demonstrated that interleukins and TNF-*α* level have been found to be high in patients with BD [43, 44]. Additionally, Hamzaoui et al. reported that vitamin D insufficiency may modulate inflammatory mediators skewing the Th1/Th2 balance towards Th1 [45]. However, it is not known whether the chronic nature of the disease and these activated inflammatory molecules affect the bone remodeling or turnover in patients with BD [5, 9, 14].

There are limited numbers of clinical trial that reveal the status of bone metabolism and bone mineral density (BMD) in the medical literature, and findings in these investigations are in consistent [8, 19, 46]. First, the status of BMD was investigated in a study, where BMD measurements of lumbar spine and hip in BD showed similar results when compared to those in healthy subjects [8]. Additionally, Tekin et al. stated that there was no significant relationship between arthritis and BMD, and bone turnover markers in patients with BD. The authors also declared that they found no statistically significant differences in BMD and bone turnover markers between BD and control groups [46]. Recently, conflicting with previous reports, Kirnap et al. have stated that they found significant differences in BMD values of lumbar spine and serum bone specific alkaline phosphatase between BD and healthy controls, indicating that BD can be a risk factor for osteoporosis in lumbar vertebra [19]. Despite these efforts, to what extent the BMD and bone remodeling in patients with BD are involved is still unknown. Further studies with larger study groups, especially relevant to bone turnover affecting the immunopathogenesis of bone metabolism and BMD in patients with BD should be considered. 

In summary, the clinical characteristics of BD are the recurrent episodes of remission and the exacerbations of various symptoms including musculoskeletal complaints. Arthritis and arthralgia are the commonest rheumatologic findings in BD. However, rare musculoskeletal findings including osteonecrosis, myalgia, and fibromyalgia may also be associated with BD [20–22]. Findings relevant to bone metabolism is lacking. Studies regarding the prevalence and characteristics of musculoskeletal findings in patients with BD are still to be investigated.

## Figures and Tables

**Table 1:** Rheumatologic findings reported in medical literature.

Findings	Localization	Percentage (%)
Joint Involvement	Arthritis/Arthralgia	40–70 [9, 14]
	Knee, wrist, ankle, elbow, hand, feet	10–56 [9, 13]
	Sacroiliac	0–63 [15, 16]
	Manubrio-sternal, sterno-clavicular	Unknown [17]
Enthesopathy	Calcaneal and vertebral bone spurs	3–36 [7, 18]
Bone Mineral Density	Vertebral osteoporosis	Unknown [19]
Muscle Involvement	Myalgia or myositis	Unknown [10, 20]
	Fibromyalgia	9.3 [21]
Bone Infarct	Osteonecrosis	Unknown [22]
